# Specializing When It Counts: Comparing the Dose–Time Effect of Distance Variety between Swimming and Track Running

**DOI:** 10.3390/sports12100272

**Published:** 2024-10-09

**Authors:** Dennis-Peter Born, Jesús J. Ruiz-Navarro, Jenny Lorentzen, Glenn Björklund

**Affiliations:** 1 Swiss Swimming Federation, Swiss Development Hub for Strength and Conditioning in Swimming, CH-3048 Worblaufen, Switzerland; 2 Department for Elite Sport, Swiss Federal Institute of Sport Magglingen, CH-2532 Magglingen, Switzerland; 3 Faculty of Science and Medicine, University of Fribourg, CH-1700 Fribourg, Switzerland; 4 Aquatics Lab, Department of Physical Education and Sports, Faculty of Sport Sciences, University of Granada, ES-18071 Granada, Spain; jesusruiz@ugr.es; 5 Swedish Winter Sports Research Centre, Mid Sweden University, SE-831 25 Östersund, Sweden; glenn.bjorklund@miun.se

**Keywords:** adolescents, diversification, elite athletes, long-term athlete development, performance, run, sampling, swim, talent

## Abstract

Objective: To conduct a longitudinal retrospective analysis, explore the relationship between success at peak performance age and the number of different race distances athletes competed in each year (within-sport distance variety), and compare the dose–time effect of this distance variety throughout the development process between male swimmers and track runners. Methodology: Male swimmers (*n* = 6033) and track runners (*n* = 19,278) still competing at peak performance age were ranked, and the number of different race distances was extracted retrospectively for each year until early junior age (13–14-year-old category) from the databases of the European Aquatics and World Athletics federations. Firstly, correlation analysis determined the relationship between ranking at peak performance age and distance variety. Secondly, Poisson distribution provided the probability and dose–time effect of distance variety for becoming an international-class athlete at peak performance age. Results: Generally, correlation analysis revealed low coefficients (*r* ≤ 0.22) but significant effects (*p* < 0.001) for larger distance variety and success at peak performance age. Poisson distribution revealed the highest probability of becoming an international-class swimmer when competing in 2–4 race distances at junior age, depending on the primary race distance. The dose–time effect indicated a gradual reduction in the number of race distances as athletes approached peak performance age, narrowing down to 1–2, 2–3, and 3–4 distances for sprint, middle-, and long-distance races, respectively. Track runners exhibited a lower distance variety than swimmers, with a consistent optimum of 1–2 race distances across the age groups. Conclusions: The present findings including data of the most combined race distances for each primary race distance and a comparison between swimming and track running provide new background information to challenge traditional training regimes and help establish new strategies for long-term athlete development.

## 1. Introduction

Should athletes focus their efforts on a single area like a laser beam, or should they spread their skills like a prism to encompass a broader range? This question is at the center of ongoing debate among talent development experts, who discuss whether athletes should concentrate on developing sport-specific skills or cultivate a wide variety of abilities to maximize their chances of success at peak performance age [1,2,3]. The controversial discussion is fueled by many recommendations being based on theoretical frameworks, a limited number of original investigations and review articles with contradictory conclusions [4,5,6,7]. Additionally, retrospective questionnaires about sports involvement during adolescence depend on the accuracy of participants’ or their guardians’ recall memories [8,9,10]. To address limitations in the recollection of subjective information, recent studies have utilized race results and objectively quantified sports involvement (within-sport variety) [11,12]. Swimming is particularly well suited for such scientific studies due to the standardized conditions under which swimming competitions are held. Specific pool length, water temperature, minimal current and environmental effects, and electronic timing make race results comparable across different competitions, venues, nations, and years [13].

Recent studies have highlighted the relationship between junior performance and later success during adulthood [11,12,14,15]. For instance, top-level 100 m freestyle swimmers began to outperform their lower-ranked peers as early as age 12 [12] and the chances of success are particularly high for swimmers who excelled at the World Junior Championships over multiple years [11]. However, the challenge in talent identification lies in recognizing athletes who do not stand out with exceptional performances during early junior years. Instead, it is crucial not to overlook late developers who exhibit a delayed yet continuous progression towards and during early adult age [16]. Particularly, a broader within-sport variety during adolescence may favor the later development of competition performances, yet promote long-term athlete development and success at peak performance age [7,17]. As such, a previous study demonstrated that within-sport variety should not be seen as an “either-or” decision, but rather as a continuous process that evolves from junior to adult age [18]. The dose–time effect revealed the highest probability of becoming an international-class swimmer when competing in a wide range of race distances during early junior age and progressively specializing towards peak performance age [18]. Since the aforementioned study only analyzed 200 m events, further research is needed to differentiate the dose–time effect among sprint, middle-, and long-distance swimmers.

While swimming is traditionally characterized by over-distance training incorporating large volumes of low-intensity training and long aerobic sets [19,20], comparison to another sport with a different training philosophy but comparable and standardized competition formats could challenge traditional training regimes in swimming. As such, track running has standardized competition formats [21,22], comparable race times [23,24], and similar physiological and metabolic demands [25,26,27,28,29,30]. Despite the similarities, track runners typically rely on high-intensity and under-distance training [31,32]. Moreover, track runners tend to have larger differences between the performances of their main and secondary race distances (quality of variety) than swimmers [33]. Yet, within a group of specialized track runners, Brustio and co-workers [34] showed that chances of success increased for runners who competed in more than one race distance. While within-sport distance variety may be a continuously evolving process and important contributor to long-term athlete development, the dose–time effect of the number of different race distances that track runners and swimmers compete in each year (quantity of variety) has not yet been explored. Analyzing races across all distances—sprint, middle-, and long-distance—and comparing the two sports could offer valuable new insights for strategic decisions in the talent development process.

Therefore, the aims of the present study were (1) to determine the relationship between success at peak performance age and the number of different race distances athletes competed in each year from junior to adult age (quantity of within-sport distance variety) and (2) to provide the dose–time effect of this distance variety throughout the long-term athlete development of male swimmers and track runners. 

## 2. Materials and Methods

### 2.1. Subjects

To conduct a longitudinal retrospective analysis of within-sport distance variety and explore the relationship between success at peak performance age and the number of different race distances athletes competed in each year throughout their development process, race times of male swimmers (*n* = 6033 from 155 countries) and track runners (*n* = 19,278 from 207 countries) were retrieved from the database of the European Aquatics [35] and World Athletics federations [36]. Since only publicly available data were included and analyzed anonymously, explicit written consent from the athletes was not required. The study protocol was preapproved by the institutional review board of the Swiss Federal Institute of Sport Magglingen (Reg.-Nr. 222_LSP_Born_03_2024) and conducted in accordance with the code of conduct of the World Medical Association for medical research involving human subjects (Declaration of Helsinki).

### 2.2. Data Collection

Only swimmers and runners still competing at peak performance age were included in the analysis. Data from the 2016 to 2023 databases were used to rank these athletes according to their personal best race times in the particular event at peak performance age. Peak performance age was set at 23 to 30 years based on previous studies [37,38] and the recently introduced U23 European Swimming Championships [39], designed as a transition phase between international junior and senior championships. To establish rankings and allow a comparison of different distances and sports, race times were converted into performance points. With swimming being the primary focus of the present study, the point system of the world governing body of swimming, which expresses race times relative to the current world record (equal to 1000 points), was applied to both swimming and track running [13].

The number of different race distances per year was retrospectively extracted back to early junior age (13–14-year-old category) for each individual athlete, also including the 2006 to 2015 databases. All freestyle long-course (50 m pool length) swimming events, i.e., 50 m, 100 m, 200 m, 400 m, 800 m, 1500 m, and events held on 400 m outdoor running tracks, i.e., 100 m, 200 m, 400 m, 800 m, 1500 m, 3000 m, 5000 m, 10,000 m were included. The “pandas” library (version 2.2.1, pandas-dev/pandas, Zenodo, Genève, Switzerland) for Python (version 3.11.5, Python Software Foundation, Beaverton, OR, USA) was used for data collection and analysis. Specifically, this software was utilized to compute performance points, establish rankings based on individual athletes’ personal bests in the specific race distances at peak performance age, and retrospectively determine the number of different race distances across the various age groups.

### 2.3. Data Analysis

The initial step involved a correlation analysis to determine the relationship between performance points of the personal best race times at peak performance age and the number of different race distances the athletes competed in each year (within-sport distance variety) across the various age categories, i.e., 13–14, 15–16, 17–18, 19–20, 21–22, and 23–30 years. This study aimed to identify contributing factors to success in high-performance sports. Therefore, swimmers and runners with a personal best of fewer than 550 performance points at peak performance age were excluded from the analysis. In the second step, the dose–time effect of distance variety throughout the development process was determined using Poisson distribution for international-class athletes, i.e., >750 performance points at peak performance age [40].

### 2.4. Statistical Analysis

All statistical analyses were conducted with the JASP statistical software package (version 0.19, JASP-Team, University of Amsterdam, Amsterdam, The Netherlands). An alpha level of 0.05 confirmed statistical significance. The relationships between performance at peak age and within-sport distance variety was assessed with Pearson’s correlation coefficient. If the Shapiro–Wilk test and Q–Q plot showed non-normally distributed data, Spearman’s rho was applied. The dose–time effect was determined by Poisson distribution, a mass function which determines the probability (*p*) of becoming an international-class athlete based on the number of different race distances an athlete competed in a particular year. Probabilities were calculated for each year from early junior age until peak performance age using Microsoft Excel 365 (version 2406, Microsoft Corporation, Redmond, WA, USA). The likelihood is expressed as a percentage and depend on the number of different race distances athletes competed in and which qre newly determined for each year [41]. Being a discrete probability mass function, the Poisson distribution represents the likelihood of an independent event, such as achieving >750 performance points at peak performance age, occurring at various time points on a constant time scale.

## 3. Results

Table 1 shows the correlation analysis between the performance points of the personal best time at peak performance age in the particular race distance and within-sport distance variety (number of different race distances the athletes competed in each year). Generally, the correlation coefficients were low and yet had highly significant effects. Swimmers showed increasing effects (*p* < 0.001), which was a larger variety in higher performance levels, towards the older age categories. Particularly, middle-distance events showed highly significant correlations between higher performance level at peak age and variety in race distances. In runners, the effects showed a U shape across the age categories, with most pronounced effects between 19 and 22 years of age. Most significant correlations (*p* < 0.001) were found in sprint and long-distance events, i.e., 100 m and 10,000 m.

**Figure 1:**
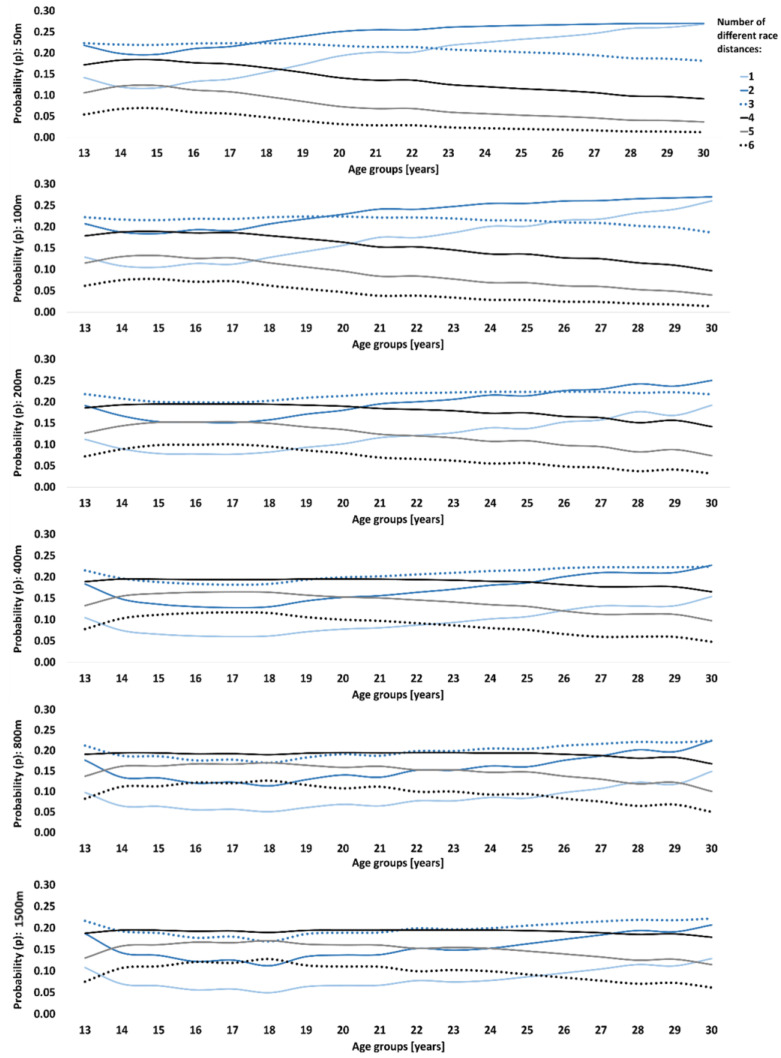
Probability (*p*) of becoming an international-class swimmer at peak performance age (>750 performance points) based on the number of different race distances (from 1 to 6) athletes competed in each age group.

During junior age, sprint swimmers (50 m and 100 m) showed the highest probability of becoming an international-class athlete when competing in three different race distances per year (Figure 1) and transitioning to two race distances at 18 and 20 years of age, respectively. Probability when competing in only one race distance increased towards peak performance age in the 50 m and 100 m events; however, it became irrelevant for longer race distances. Variety was generally larger with longer race distances. As such, 400 m, 800 m, and 1500 m swimmers benefited from four different race distances during junior age. The transition point to three race distances occurred at an older age with longer race distances, i.e., at 19 years of age in 400 m vs. 21–22 years of age in 800 m and 1500 m. Table 2 shows the most combined race distances across the various age categories. At peak performance age, 50 m events were mostly combined with 100 m, 100 m with 50 m and 200 m, and 200 m with 50 m and 100 m. The 400 m swimmers competed across all race distances, i.e., 50 m to 1500 m. The 800 m and 1500 m swimmers competed in race distances of 200 m and longer.

**Figure 2:**
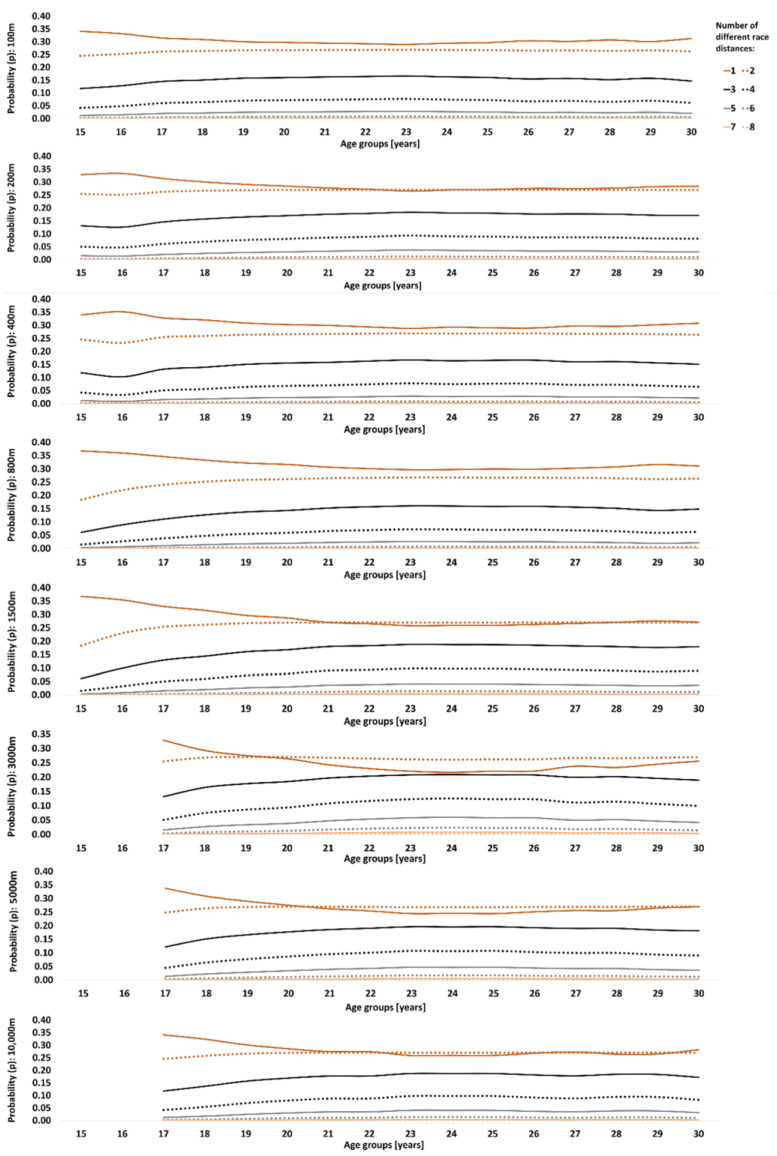
Probability (*p*) of becoming an international-class track runner at peak performance age (>750 performance points) based on the number of different race distances (from 1 to 8) athletes competed in each age group.

Track runners showed lower variety compared to swimmers (Figure 2). Competing in only one race distance showed the highest probability of becoming an international-class athlete at peak performance age for 100 m, 200 m, 400 m, and 800 m. For longer race distances, competing in one or two race distances showed equally high probabilities. The most common combined race distances are shown in Table 3. At peak performance age, the 100 m and 200 m events were commonly combined. The 400 m runners competed across all sprint distances of track running, i.e., 100 m to 400 m. The 800 m and 1500 m events were commonly combined with each other, and so were the 5000 m and 10,000 m events. The 3000 m runners commonly competed in 1500 m to 10,000 m events.

## 4. Discussion

The present study investigated the quantity of variety in regard to correlations between the number of different race distances athletes competed in each year and their performance at peak performance age. Additionally, the dose–time effect of within-sport distance variety and probability to become an international-class athlete was determined with Poisson distribution. Given that many key performance indicators in swimming rely on technical skills rather than physiological capacities, swimmers often specialize in a particular stroke rather than a specific race distance [42]. With up to six race distances in freestyle events [21], swimmers can maximize their chances of winning medals by successfully transferring their technical skills across various race distances. This helps explain why swimmers tend to have a greater variety of race distances compared to track runners, who rely more on physiological performance indicators [43,44]. However, differences in distance variety exist, depending on the primary race distance athletes competed in at peak performance age. For instance, 400 m swimmers commonly competed across the full range of distances (from 50 m to 1500 m) at peak performance age. The 400 m freestyle events sit in the middle of the distance spectrum and offer fewer options for different swimming strokes compared to 100 m and 200 m races, which are also held for the butterfly, backstroke, breaststroke, and individual medley. Therefore, 100 m and 200 m swimmers can compete in various swimming strokes at their preferred race distance, such as butterfly and backstroke swimmers commonly also competing in freestyle [18]. The smaller number of different 400 m events, which are only held for freestyle and individual medley, may explain the larger distance variety and contribute to the fact that 400 m specialists also compete in all the other race distances (refer to Table 2)

The present study showed a smaller distance variety for freestyle sprint compared to middle- and long-distance swimmers. As such, 45.5% of the 50 m freestyle swimmers commonly focused on the 50 m and 100 m events. In addition to the need for high anaerobic energy production [30], effectively catching water at stroke rates as high as 61.9 ± 1.8 strokes/min and translating them into sprint swimming velocities of 2.12 ± 0.02 m/s underlies a specific technical element of sprint races [45,46,47]. This is particularly difficult at higher velocities, since water does not provide a solid resistance from which swimmers’ hands can push. Instead, swimmers have to catch the water and progressively build up the resistance for each arm stroke, while minor changes in joint angles and hand positioning can significantly impact each arm stroke’s efficiency and propulsion [48,49]. These technical elements result in a different swimming technique with the characteristic straight arm during the overwater phase, which may not be easily learned for a middle- or long-distance swimmer, and may therefore explain the earlier and higher degree of specialization in sprint swimmers. Furthermore, the importance of the acyclic phases in sprint races underscores the need for neuromuscular skills, such as explosive leg strength [47,50,51,52,53]. Since these skills are primarily developed through dry-land training and complex weightlifting exercises [47,50,51], earlier and more specific practice seems necessary to learn and refine these abilities and may contribute to the lower distance variety among sprint swimmers.

Traditional training regimes may also explain the larger distance variety in swimming than track running. Swimmers typically emphasize over-distance training and large volumes of aerobic work [19,20]. A generally large exposure to the specific competition environment, i.e., water, appears to help develop the necessary technical skills—which coaches and swim experts often refer to as a “feeling for the water”—required for in-water locomotion [20]. The larger training volume compared to runners may be important for swimmers, since humans are evolutionarily land-based mammalians and unaccustomed to water. Additionally, due to the large distance variety, swimmers compete in multiple heats and finals during a single competition day. Here, a large aerobic capacity helps in quick recovery between high-intensity efforts [54]. In contrast, track runners focus more on high-intensity and under-distance training specific to their primary race distance [32,43]. The different training approaches in runners are influenced by the injury risks associated with the impact forces of running [55,56,57]. Although swimmers are not exposed to the injury risk of weight-bearing exercise, their development of neuromuscular abilities and anaerobic energy contribution may also benefit by the incorporation of more race–pace-specific bouts and dry-land practice [20,30,51,58].

The greater number of countries and athletes involved in track running compared to swimming (207 vs. 155 in the present sample) may contribute to a higher performance density in track events, compelling athletes to specialize and pinpointing their physiological adaptations for specific events. Although performance density across different sports and disciplines warrants a comprehensive investigation, the present comparison to track running suggests that swimmers may not have yet fully reached their physiological potential and higher specialization in specific race distances when approaching peak performance age may further develop their race times. However, the reader must also keep in mind that multiple factors contribute to the development of competition performance from early junior to adult age [59]. While anthropometric, socioeconomic, and infrastructural factors are difficult to change or require a substantial amount of time to apply any adjustments, coaches can easily adapt within-sport distance variety to meet the specific demand of their sprint, middle-, and long-distance swimmers.

## 5. Conclusions

The investigation of the quantity of variety, i.e., the number of different race distances athletes competed in each year, revealed a greater within-sport distance variety for swimmers compared to runners. While correlation analysis indicated that a larger variety was associated with success at peak performance age, probability analysis demonstrated that this variety had a dose–time effect specific to the primary race distance and age group. The highest probability of becoming an international-class athlete was observed with greater variety during junior age, followed by progressive specialization as athletes approached peak performance age. Based on the comparison of track running and swimming, an earlier and more focused approach to specific race distances could potentially lead to further improvements in swimming race times.

## Figures and Tables

**Table 1:** Correlation analysis between the performance at peak age (in the respective race distance) and number of different race distances across the various age categories. The correlation analysis included world-class finalists, international-, national-, and regional-class swimmers, or runners (>550 performance points at peak performance age).

	Age Categories (Years)					
	13–14	15–16	17–18	19–20	21–22	23+
**Swimmers**						
50 m	0.06 *	0.00	0.00	0.00	0.05 **	0.10 ***
100 m	0.08 ***	0.02	0.04 *	0.04 *	0.10 ***	0.14 ***
200 m	0.10 ***	0.06 **	0.06 **	0.11 ***	0.14 ***	0.13 ***
400 m	0.09 **	0.12 ***	0.13 ***	0.17 ***	0.19 ***	0.17 ***
800 m	0.07	0.06	0.06	0.13 ***	0.11 **	0.05
1500 m	0.10	0.04	0.03	0.09 *	0.08 *	0.01
**Runners**						
100 m		0.02	0.13 ***	0.10 ***	0.09 ***	0.05 *
200 m		0.09	0.18 ***	0.16 ***	0.07 *	0.10 ***
400 m		−0.04	0.06	0.05	0.05	0.05
800 m		−0.04	0.08 *	0.13 ***	0.12 ***	0.01
1500 m		0.03	0.11 **	0.09 **	0.10 ***	0.08 **
3000 m		0.22	0.10	0.06	0.03	0.07
5000 m		0.15	0.10	0.14 ***	0.07 *	0.13 ***
10,000 m		0.00	0.22 ***	0.16 ***	0.19 ***	0.09 *

Statistical significance: * *p* < 0.05, ** *p* < 0.01, *** *p* < 0.001.

**Table 2:** Most combined race distances of international-class (>750 performance points at peak performance age) 50 m, 100 m, 200 m, 400 m, 800 m, and 1500 m swimmers across the various age categories.

	Age Categories [Years]					
	13–14	15–16	17–18	19–20	21–22	23+
**50 m**	**30.6% ^[50, 100, 200, 400]^** 23.1% ^[50, 100, 200]^ 22.5% ^[50, 100, 200, 400, 800, 1500]^	**33.6% ^[50, 100, 200]^** 24.9% ^[50, 100, 200, 400]^ 17.2% ^[50, 100, 200, 400, 800, 1500]^	**40.0% ^[50, 100, 200]^** 24.4% ^[50, 100, 200, 400]^ 22.4% ^[50, 100]^	**45.7% ^[50, 100, 200]^** 31.4% ^[50, 100]^ 13.3% ^[50, 100, 200, 400]^	**43.6% ^[50, 100, 200]^** 37.2% ^[50, 100]^ 10.6% ^[50, 100, 200, 400]^	**45.5% ^[50, 100]^** 37.0% ^[50, 100, 200]^ 8.5% ^[50, 100, 200, 400]^
**100 m**	**24.8% ^[50, 100, 200, 400]^** 22.5% ^[50, 100, 200, 400, 800, 1500]^ 14.8% ^[50, 100, 200]^	**24.3% ^[50, 100, 200]^** 22.7% ^[50, 100, 200, 400]^ 22.4% ^[50, 100, 200, 400, 800, 1500]^	**30.3% ^[50, 100, 200]^** 25.0% ^[50, 100, 200, 400]^ 15.7% ^[50, 100, 200, 400, 800, 1500]^	**35.4% ^[50, 100, 200]^** 19.6% ^[50, 100, 200, 400]^ 18.2% ^[50, 100]^	3**8.4% ^[50, 100, 200]^** 24.4% ^[50, 100]^ 14.8% ^[50, 100, 200, 400]^	**37.2% ^[^^50, 100, 200]^** 30.8% ^[50, 100]^ 12.9% ^[50, 100, 200, 400]^
**200 m**	**31.5% ^[50, 100, 200, 400, 800, 1500]^** 21.1% ^[50, 100, 200, 400]^ 12.8% ^[100, 200, 400, 800, 1500]^	**33.8% ^[50, 100, 200, 400, 800, 1500]^** 16.0% ^[50, 100, 200, 400]^ 14.6% ^[100, 200, 400, 800, 1500]^	3**0.3% ^[50, 100, 200, 400, 800, 1500]^** 22.0% ^[50, 100, 200, 400]^ 15.4% ^[50, 100, 200]^	**21.1% ^[50, 100, 200, 400]^** 20.0% ^[50, 100, 200, 400, 800, 1500]^ 19.4% ^[50, 100, 200]^	**22.1% ^[50, 100, 200]^** 21.9% ^[50, 100, 200, 400]^ 12.3% ^[50, 100, 200, 400, 800, 1500]^	**25.0% ^[^^50, 100, 200]^** 22.3% ^[50, 100, 200, 400]^ 11.1% ^[50, 100, 200, 400, 800, 1500]^
**400 m**	**37.8% ^[50, 100, 200, 400, 800, 1500]^** 16.8% ^[100, 200, 400, 800, 1500]^ 15.9% ^[50, 100, 200, 400]^	**40.7% ^[50, 100, 200, 400, 800, 1500]^** 19.6% ^[100, 200, 400, 800, 1500]^ 10.8% ^[50, 100, 200, 400]^	**38.0% ^[50, 100, 200, 400, 800, 1500]^** 21.1% ^[100, 200, 400, 800, 1500]^ 12.1% ^[50, 100, 200, 400]^	**24.3% ^[50, 100, 200, 400, 800, 1500]^** 18.1% ^[200, 400, 800, 1500]^ 15.2% ^[100, 200, 400, 800, 1500]^	**18.8% ^[100, 200, 400, 800, 1500]^** 18.3% ^[50, 100, 200, 400, 800, 1500]^ 18.1% ^[200, 400, 800, 1500]^	**18.1% ^[50, 100, 200, 400, 800, 1500]^** 17.5% ^[50, 100, 200, 400]^ 15.0% ^[200, 400, 800, 1500]^
**800 m**	**49.4% ^[50, 100, 200, 400, 800, 1500]^** 17.2% ^[100, 200, 400, 800, 1500]^ 11.8% ^[200, 400, 800, 1500]^	**41.8% ^[50, 100, 200, 400, 800, 1500]^** 27.9% ^[100, 200, 400, 800, 1500]^ 15.6% ^[200, 400, 800, 1500]^	**40.4% ^[50, 100, 200, 400, 800, 1500]^** 29.2% ^[100, 200, 400, 800, 1500]^ 18.0% ^[200, 400, 800, 1500]^	**30.8% ^[200, 400, 800, 1500]^** 25.5% ^[50, 100, 200, 400, 800, 1500]^ 20.7% ^[100, 200, 400, 800, 1500]^	**31.7% ^[200, 400, 800, 1500]^** 22.1% ^[100, 200, 400, 800, 1500]^ 21.3% ^[50, 100, 200, 400, 800, 1500]^	**26.4% ^[200, 400, 800, 1500]^** 22.6% ^[50, 100, 200, 400, 800, 1500]^ 18.8% ^[400, 800, 1500]^
**1500 m**	**56.1% ^[50, 100, 200, 400, 800, 1500]^** 20.5% ^[100, 200, 400, 800, 1500]^ 12.3% ^[400, 800, 1500]^	**42.3% ^[50, 100, 200, 400, 800, 1500]^** 28.4% ^[100, 200, 400, 800, 1500]^ 13.9% ^[200, 400, 800, 1500]^	**38.4% ^[50, 100, 200, 400, 800, 1500]^** 30.2% ^[100, 200, 400, 800, 1500]^ 22.0% ^[200, 400, 800, 1500]^	**37.9% ^[200, 400, 800, 1500]^** 25.0% ^[50, 100, 200, 400, 800, 1500]^ 19.7% ^[100, 200, 400, 800, 1500]^	**38.6% ^[200, 400, 800, 1500]^** 23.2% ^[100, 200, 400, 800, 1500]^ 19.5% ^[50, 100, 200, 400, 800, 1500]^	**33.6% ^[200, 400, 800, 1500]^** 25.2% ^[400, 800, 1500]^ 24.4% ^[50, 100, 200, 400, 800, 1500]^

**Table 3:** Most combined race distances of international-class (>750 performance points at peak performance age) 100 m, 200 m, 400 m, 800 m, 1500 m, 3000 m, 5000 m, and 10,000 m track runners across the various age categories.

	Age Categories [Years]				
	15–16	17–18	19–20	21–22	23+
**100 m**	**59.0% ^[100, 200]^** 38.0% ^[100]^ 3.0% ^[100, 200, 400]^	**65.8% ^[100, 200]^** 23.6% ^[100]^ 10.5% ^[100, 200, 400]^	**68.1% ^[100, 200]^** 16.9% ^[100]^ 14.7% ^[100, 200, 400]^	**68.5% ^[100, 200]^** 15.9% ^[100]^ 15.4% ^[100, 200, 400]^	**62.0% ^[100, 200]^** 24.4% ^[100, 200, 400]^ 13.2% ^[100]^
**200 m**	**51.4% ^[100, 200]^** 30.1% ^[200]^ 12.5% ^[200, 400]^	**59.0% ^[100, 200]^** 14.7% ^[200, 400]^ 14.2% ^[100, 200, 400]^	**57.6% ^[100, 200]^** 20.2% ^[100, 200, 400]^ 15.5% ^[200, 400]^	**57.1% ^[100, 200]^** 23.8% ^[100, 200, 400]^ 14.5% ^[200, 400]^	**50.0% ^[100, 200]^** 37.8% ^[100, 200, 400]^ 9.1% ^[200, 400]^
**400 m**	**67.5% ^[400]^** 23.1% ^[200, 400]^ 5.6% ^[100, 200, 400]^	**46.9% ^[400]^** 28.1% ^[200, 400]^ 15.5% ^[100, 200, 400]^	**38.5% ^[200, 400]^** 28.3% ^[400]^ 22.4% ^[100, 200, 400]^	**40.7% ^[200, 400]^** 25.1% ^[400]^ 24.6% ^[100, 200, 400]^	**38.1% ^[100, 200, 400]^** 33.8% ^[200, 400]^ 15.8% ^[400]^
**800 m**	**76.7% ^[800]^** 13.6% ^[400, 800]^ 8.2% ^[800, 1500]^	**50.9% ^[800]^** 20.2% ^[800, 1500]^ 18.4% ^[400, 800]^	**33.8%**^**[800]**^ 30.7% ^[800, 1500]^ 19.2% ^[400, 800]^	**35.0% ^[800, 1500]^** 26.4% ^[800]^ 19.6% ^[400, 800]^	**31.4% ^[800, 1500]^** 19.0% ^[400, 800, 1500]^ 16.5% ^[400, 800]^
**1500 m**	**53.1% ^[1500]^** 25.0% ^[800, 1500]^ 15.6% ^[1500, 3000]^	**37.9% ^[800, 1500]^** 22.3% ^[1500]^ 12.1% ^[800, 1500, 3000]^	**43.8% ^[800, 1500]^** 17.2% ^[1500]^ 10.1% ^[800, 1500, 3000]^	**40.1% ^[800, 1500]^** 16.3% ^[1500]^ 10.5% ^[800, 1500, 3000]^	**26.3% ^[800, 1500]^** 15.7% ^[800, 1500, 3000, 5000]^ 12.9% ^[1500, 3000, 5000, 10,000]^
**3000 m**		**21.6% ^[1500, 3000, 5000]^** 21.6% ^[3000]^ 14.1% ^[800, 1500, 3000]^	**23.1% ^[1500, 3000, 5000]^** 15.5% ^[800, 1500, 3000]^ 14.5% ^[800, 1500, 3000, 5000]^	**21.3% ^[1500, 3000, 5000]^** 20.9% ^[1500, 3000, 5000, 10,000]^ 12.5% ^[800, 1500, 3000]^	**34.3% ^[1500, 3000, 5000, 10,000]^** 16.3% ^[800, 1500, 3000, 5000]^ 14.0% ^[1500, 3000, 5000]^
**5000 m**		**38.4% ^[5000]^** 17.6% ^[3000, 5000]^ 16.4% ^[1500, 3000, 5000]^	**31.0% ^[5000, 10,000]^** 15.9% ^[1500, 3000, 5000]^ 15.9% ^[5000]^	**33.9% ^[5000, 10,000]^** 15.8% ^[1500, 3000, 5000, 10,000]^ 15.0% ^[1500, 3000, 5000]^	**28.7% ^[5000, 10,000]^** 28.4% ^[1500, 3000, 5000, 10,000]^ 11.2% ^[3000, 5000, 10,000]^
**10,000 m**		**47.2% ^[5000, 10,000]^** 32.7% ^[10,000]^ 12.7% ^[3000, 5000, 10,000]^	**49.6% ^[5000, 10,000]^** 29.3% ^[10,000]^ 13.9% ^[3000, 5000, 10,000]^	**54.1% ^[5000, 10,000]^** 18.7% ^[10,000]^ 13.4% ^[1500, 3000, 5000, 10,000]^	**48.3% ^[5000, 10,000]^** 25.4% ^[1500, 3000, 5000, 10,000]^ 15.8% ^[3000, 5000, 10,000]^

## Data Availability

The data used in this study are available on request by the corresponding author and freely accessible at swimrankings.net, the Stats Zone of the World Athletics federation (https://worldathletics.org/stats-zone), and https://doi.org/10.5281/zenodo.13773073.
